# Association between Periodontitis Extent, Severity, and Progression Rate with Systemic Diseases and Smoking: A Retrospective Study

**DOI:** 10.3390/jpm13050814

**Published:** 2023-05-11

**Authors:** Georgios S. Chatzopoulos, Ziou Jiang, Nicholas Marka, Larry F. Wolff

**Affiliations:** 1 Division of Periodontology, Department of Developmental and Surgical Sciences, School of Dentistry, University of Minnesota, Minneapolis, MN 55455, USA; wolff001@umn.edu; 2 Department of Preventive Dentistry, Periodontology and Implant Biology, Faculty of Dentistry, School of Health Sciences, Aristotle University of Thessaloniki, 54124 Thessaloniki, Greece; 3 Biostatistical Design and Analysis Center, Clinical and Translational Science Institute, University of Minnesota, Minneapolis, MN 55414, USA; jian0717@umn.edu (Z.J.); marka007@umn.edu (N.M.)

**Keywords:** epidemiology, oral health, oral-systemic disease, periodontal disease, tooth loss

## Abstract

Background: The aim of this study was to analyze the relationship between extent, severity (stage), and rate of progression (grade) of periodontitis with systemic diseases as well as smoking using a large database. Methods: Patients’ records identified in the BigMouth Dental Data Repository with a periodontal diagnosis based on the 2017 World Workshop on the Classification of Periodontal and Peri-Implant Diseases and Conditions were evaluated. Patients were further categorized based on extent, severity, and rate of progression. Data were extracted from patients’ electronic health records including demographic characteristics, dental procedural codes, and self-reported medical conditions, as well as the number of missing teeth. Results: A total of 2069 complete records were ultimately included in the analysis. Males were more likely to have generalized periodontitis and stage III or IV periodontitis. Older individuals were more likely diagnosed with grade B and stage III or IV periodontitis. Individuals with generalized disease, grade C, and stage IV demonstrated a significantly higher number of missing teeth. Higher numbers of tooth loss reported during supportive periodontal treatment were noted in generalized disease and stage IV periodontitis. Multiple sclerosis and smoking were significantly associated with grade C periodontitis. Conclusions: Within the limitations of this retrospective study that utilized the BigMouth dental data repository, smokers were significantly associated with rapid progression of periodontitis (grade C). Gender, age, number of missing teeth, and number of tooth loss during supportive periodontal treatment were associated with disease characteristics.

## 1. Introduction

Periodontal disease is a chronic inflammatory disease associated with a multifactorial etiology and characterized by the presence of dysbiotic microbial biofilms [1]. It affects the tooth-supporting tissues, including gingiva, periodontal ligaments, and alveolar bones, when the periodontal microbiome and the host response are not balanced, and it can lead to tooth loss if left untreated [1]. Periodontal disease is highly prevalent, and it has been shown that 42.2% of US dentate adults over 30 years old have periodontitis, while older individuals, specific races, and smokers demonstrate a higher risk of being diagnosed with it [2]. The pathogenesis of periodontal disease has been extensively investigated, and this has led to the development of classifications systems.

Initially, in 1976, Page and Schroeder classified periodontal diseases into four stages based on the presence of histological (initial lesion) or clinical (early lesion) signs of inflammation, changes in the color and contour of the gingiva, and the presence of gingival bleeding (established lesion), or ultimately, attachment loss (advanced lesion) [3]. At the 1989 World Workshop in Clinical Periodontics, periodontitis was classified based on the disease progression [4]. In 1999, Armitage, in collaboration with the American Academy of Periodontology, introduced a new classification system that considered disease severity and extent and categorized periodontitis into slight, moderate, and severe, as well as generalized or localized [5]. Age restrictions in aggressive periodontitis and the introduction of the term “reduced periodontium” were later used by a task force of the American Academy of Periodontology in 2015 [6]. 

Recently, in 2017, a new classification system was developed according to the current evidence which is based on a multidimensional staging and grading system [7]. Staging describes the extent and distribution of periodontitis and depends on the severity and complexity of the disease expressed by the severity of attachment loss and radiographic bone loss [7]. Grading highlights the rate of periodontitis progression, and can be used to stratify patients based on the risk of disease progression considering the systemic health and smoking status of the individuals, among others. It is of paramount importance to use existing databases to implement and validate the new classification system [8].

Oral diseases, including periodontitis and caries, are associated with a variety of systemic conditions [9]. Periodontitis negatively affects systemic health through biologically plausible mechanisms, whereas periodontal treatment may reduce the risk of disease comorbidities [9]. Bacteria associated with periodontal disease may disseminate to various tissues, causing inflammatory and functional complications, including endothelial cell dysfunction, bone marrow alterations, gut dysbiosis, and immune suppression, that may initiate or worsen comorbid pathologies. In addition, the inflammatory adaptation of hematopoietic progenitors in the bone marrow may explain the connection between multiple chronic inflammatory diseases. A systematic mapping of trial registers reported that periodontal disease is associated with 57 systemic conditions, which represents about 2% of the diseases indexed in Medical Subject Heading (MeSH) terms [10]. The potential causal relationships between oral diseases and systemic diseases may be explained by inflammatory mechanisms or may be a result of a disruptive host immune response [11]. It is also possible that susceptible hosts, due to the presence of systemic conditions or history of smoking, can shift the polymicrobial synergy towards dysbiosis, which leads to periodontitis onset and progression [12]. Hence, large-scale studies assessing the associations between periodontitis and other chronic diseases are needed. Identifying systemic diseases that may serve as risk factors or risk indicators of periodontitis during medical or dental examinations can facilitate the development of individualized interdisciplinary treatment plans [13]. 

Earlier studies evaluating the associations between systemic diseases and periodontitis are based on a periodontal classification system including slight, moderate, and severe periodontitis. The new periodontal classification, which classifies the severity and extent of the condition as well as the rate of disease progression, can be used to study how staging, grading, and disease extent are associated with systemic medical conditions and smoking. Therefore, the aim of this study was to analyze the relationship between extent, severity (stage), and rate of progression (grade) of periodontitis with systemic diseases as well as smoking. Using a large database such as the BigMouth Dental Data Repository allows generalizability across multiple dental institutions, plays a key role in developing preventative care models, and may assist practitioners in providing a more accurate and enhanced treatment.

## 2. Materials and Methods

This cross-sectional study was reviewed by the Institutional Review Board of the University of Minnesota and it was determined that it is not research involving human subjects, as defined by the Department of Health and Human Services as well as the United States Food and Drug Administration (STUDY00016576). It was further reviewed and approved by the BigMouth Consortium for Oral Health Research and Informatics clinical review committee. This study was conducted in agreement with the Helsinki Declaration of 1975 as most recently revised in 2013.

The BigMouth Dental Data Repository was utilized to extract electronic health records from 2011–2021. Dental charts of adult patients who had attended the dental clinics seeking dental therapy of the universities contributing data to the BigMouth network and accepted the protocol of the study were evaluated: Harvard University; University of Texas Health; The University of California, San Francisco; University of Colorado; Loma Linda University; University of Buffalo; The University of Iowa; The University of Minnesota. Electronic health records were entered during patients’ visits by both dental students, residents, and faculty oral health care providers. Dental Procedure Codes and Current Procedural Terminology procedures were utilized. Current Dental Terminology (CDT) is a code set with descriptive terms developed and updated by the American Dental Association (ADA) for reporting dental services and procedures to dental benefits plans. Patients with at least one completed treatment code D0150 (comprehensive Oral Evaluation), D0120 (periodic oral evaluation provided to an established patient), or D0180 (comprehensive restorative and periodontal exam) were included.

Patients’ records identified in the BigMouth Dental Data Repository with a periodontal diagnosis based on the 2017 World Workshop on the Classification of Periodontal and Peri-Implant Diseases and Conditions were evaluated [7,8]. Patients were further categorized into the following categories based on the extent, severity, and rate of progression:

A. Extent of periodontitis:

(i) Localized (<30% of teeth involved)
(ii) Generalized (>30% of teeth involved)

B. Severity of periodontitis:

(i) Stage I (interdental clinical attachment loss of 1–2 mm and radiographic bone loss < 15%)
(ii) Stage II (interdental clinical attachment loss of 3–4 mm and radiographic bone loss 15–33%)
(iii) Stage III (interdental clinical attachment loss of ≥5 mm, radiographic bone loss extending to the middle third of root and beyond, and showed ≤4 teeth lost due to periodontitis)
(iv) Stage IV (interdental clinical attachment loss of ≥5 mm, radiographic bone loss extending to the middle third of root and beyond, and showed ≥5 teeth lost due to periodontitis)

C. Rate of progression:

(i) Grade A—slow rate (no bone loss or attachment loss over five years or percentage of bone loss/age <0.25, nonsmoking, no diagnosis of diabetes)
(ii) Grade B—moderate rate (<2 mm bone loss or attachment loss over five years or percentage of bone loss/age 0.25–1.0, smoking <10 cigarettes/day, and HbA1c < 7.0% in diabetics)
(iii) Grade C—rapid rate (≥2 mm bone loss or attachment loss over 5 years or percentage of bone loss/age > 1, smoking ≥10 cigarettes/day, and HbA1c ≥ 7.0% in patients with diabetes)

Other relevant data were extracted from patients’ electronic records, including demographic characteristics, patient-reported medical conditions, and number of missing teeth prior to the diagnosis, as well as tooth loss during the periodontal maintenance. Demographic characteristics included: age (at baseline); ethnicity (Hispanic, non-Hispanic, other); race (Asian, African American, Pacific islander, American/Indian/Alaskan native, white, Hispanic or Latino, other); gender (male, female). In addition, patient-reported medical conditions included the following: alcohol consumption, blood and hematological disorders, cancer, angina, congenital heart disease, coronary heart disease, history of endocarditis, heart attack, high blood pressure, implant defibrillator, rheumatic fever, smoking, cocaine use, marijuana use, adrenal gland disorder, diabetes, thyroid disorders, glaucoma, HIV, sexually transmitted disease, dialysis, kidney disease, renal failure or insufficiency, arthritis, lupus, osteoporosis, dementia, anxiety, depression, Parkinson disease, multiple sclerosis, seizure or epilepsy, stroke, organ transplantation, asthma, chronic bronchitis or emphysema, sleep apnea, Crohn’s disease, gastrointestinal disorder, hepatitis. Self-reported systemic diseases are based on a patient questionnaire that is completed at the initial visit and updated every 6–12 months.

### Statistical Analysis

Patients’ demographic and clinical characteristics were summarized as means and standard deviations or medians and interquartile ranges (IQRs) for continuous variables as appropriate, and frequencies and percentages for categorical variables. Normality was assessed using density plots. Between generalized and localized periodontal disease patients, two sample t, Wilcoxon Rank Sum, and Chi-square tests were used for comparison of normally distributed, nonnormally distributed, and categorical outcomes, respectively. Among different rates of progression and periodontitis disease severities, ANOVA, Kruskall-Wallis, and Chi-square tests were used for comparison of normally distributed, nonnormally distributed, and categorical outcomes, respectively. Associations between systemic medical conditions and periodontal disease extent were assessed using a logistic regression model with results presented as Odds Ratios (OR) and their corresponding 95% confidence intervals (CI). Medical conditions’ associations with periodontal disease progression rate and disease severity were both assessed using proportional odds regression models, with results presented as Odds Ratios (OR) and their corresponding 95% CI. Proportional odds assumptions were assessed using the Brant test. Multivariable regression models were explored for the above outcomes, adjusting for the effects of age, gender, and missing teeth at baseline. The *p*-values were corrected for multiple comparisons using the Holm method. All analyses were based on complete cases and were conducted at the 0.05 significance level using the R environment (Version 4.2.1). More specifically, the following packages were utilized: (i) version 4.2.1 (use for binomial regression, *p* value adjustment, Wilcoxon rank sum test, Chi-square test, density); (ii) base package, version 4.2.1 (used for calculating means, sd, median, IQR); (iii) MASS package, version 7.3-57 (used for negative binomial regression).

## 3. Results

A total of 164,892 records of patients who had periodontal treatment at any of the eight included US universities between 2011–2021 were identified in the BigMouth Dental Data Repository using the inclusion and exclusion criteria of the study. After screening the periodontal diagnoses for all potentially eligible individuals, 2807 patient records were identified having used the new Classification of Periodontal and Peri-Implant Diseases and have categorized each patient into the three parameters of interest (extent, stage, and grade). The analysis performed in this study is based on 2069 complete records after excluding records with missing data. The distribution of the included population was: females (56.2%), Hispanic ethnic group (67.7%), Hispanic race (34.8%), and their mean age was 53.81 ± 14.13 years.

### 3.1. Periodontal Disease Extent

The demographic characteristics of the included population stratified by disease extent are shown in Table 1. Overall, the extent of periodontal disease was almost equally divided into this patients’ cohort (generalized n = 1043 and localized n = 1026). Univariable comparison tests indicated significant difference in gender, missing teeth, and tooth loss during periodontal maintenance between the two extent levels. The generalized periodontitis group had significantly higher proportions of males (*p* = 0.022) as well as a higher number of missing teeth at baseline (*p* = 0.001) and tooth loss as reported during the periodontal maintenance visit (*p* = 0.012). The association between systemic medical conditions and periodontal disease extent is demonstrated in Table 2. Multivariable logistic regressions, adjusted for age, gender, and number of missing teeth, showed that none of the examined systemic conditions are significantly associated with periodontal disease extent. 

### 3.2. Periodontal Disease Progression

The demographic characteristics of the included population stratified by periodontal disease grade are shown in Table 3. The majority of the included population was diagnosed with Grade B periodontitis (n = 1403). Univariable comparison tests indicated significant difference in age and the number of missing teeth at baseline between Grades A, B, and C. Individuals in the Grade B periodontitis group were significantly older (*p* = 0.032), while patients with Grade C periodontitis exhibited significantly higher numbers of missing teeth (*p* = 0.019). None of the other demographic characteristics reached the level of significance. The association between systemic medical conditions and periodontal disease rate of progression (grade) is demonstrated in Table 4. The multivariable proportional odds regressions, adjusted for age, gender, and the number of missing teeth revealed that smoking (*p* = 0.014) and multiple sclerosis (*p* < 0.001) are significantly associated with the rate of progression. Individuals with multiple sclerosis and smoking habits were significantly more likely to have higher-grade periodontitis. 

### 3.3. Periodontal Disease Severity

The demographic characteristics of the included population stratified by periodontal disease severity (stage) are shown in Table 5. Most of the included periodontitis patients were diagnosed with stage III periodontitis, while only 138 individuals exhibited stage IV. Univariable comparison tests indicated significant difference in age (*p* < 0.001), gender (*p* = 0.004), number of missing teeth (*p* < 0.001), and tooth loss as reported during the periodontal maintenance visit (*p* < 0.001) among the four periodontitis stages. Older individuals and males were more likely to have stage III and IV periodontitis. In addition, stage IV periodontitis patients showed a significantly higher number of missing teeth prior to periodontal treatment and lost more teeth than individuals with less severe disease. The association between systemic medical conditions and periodontal disease severity (stage) is demonstrated in Table 6. Multivariable proportional odds regressions adjusted for age, gender, and the number of missing teeth showed that none of the examined systemic conditions in this population group are significantly associated with periodontal disease severity.

## 4. Discussion

**Figure 1:**
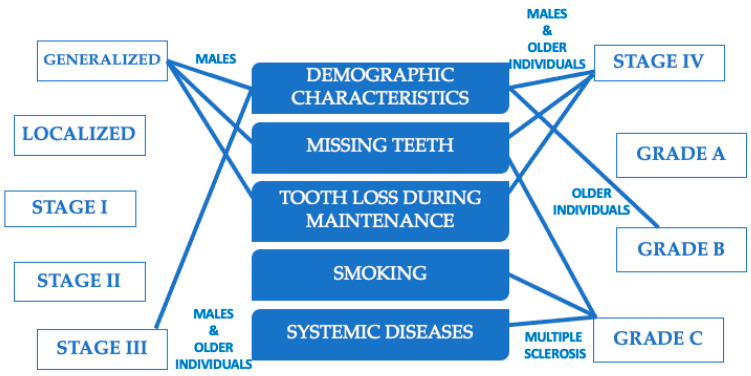
Summary of this study’s main findings.

Findings regarding the associations between systemic medical conditions and smoking with periodontal disease are solely based on the previous periodontal classification. In this retrospective investigation, 2069 patients who attended eight university dental clinics in the US seeking dental therapy were included to assess the relationship between extent, severity (stage), and rate of progression (grade) of periodontitis with systemic disease and smoking using the BigMouth Dental Data Repository. To the best of our knowledge, this is the first investigation to assess this aim using the new periodontal classification. The results of the present study indicated that: (i) males were more likely to have generalized periodontitis and stage III or IV periodontitis; (ii) older individuals were more likely diagnosed with grade B and stage III or IV periodontitis; (iii) individuals with generalized disease, grade C and stage IV demonstrated a significantly higher number of missing teeth; (iv) higher numbers of tooth loss reported during supportive periodontal treatment were noted in generalized disease and stage IV periodontitis; (v) multiple sclerosis and smoking were significantly associated with grade C periodontitis. This study’s findings are also summarized in Figure 1. The results of the present study confirmed the validity of the 2017 Classification of Periodontal and Peri-Implant Diseases and Conditions, as it was shown that individuals with generalized and stage IV periodontitis demonstrated a greater teeth loss during supportive phase of periodontal treatment. Moreover, the study confirmed the association of smoking with rapid progression (Grade C) of periodontal disease.

Gender is one of the most investigated risk factors associated with periodontitis in the periodontal literature. Although different studies have demonstrated that males are more prone to develop periodontal disease than females, no clear relationship has been identified. It has been assumed that males exhibit worse oral hygiene and less frequent professional care than females, which explains the increased biofilm accumulation and therefore disease onset and progression [14]. From an epidemiological standpoint, reports based on the NHANES data from the US have shown that males demonstrate a higher prevalence of severe periodontitis [15]. This is in agreement with the findings of the present study. A systematic review of the literature has also clearly reported that males exhibit a greater prevalence and severity of periodontal disease than females of comparable age [14]. It is unclear whether there is a biologic basis for this difference, or if gender can be a factor that modifies the pathogenesis and progression of periodontitis as it happens in other conditions [16].

The prevalence of periodontal disease is gradually increasing with age, and this might be due to the cumulative effect of the bacteria over time [15]. Older individuals included in the present investigation were more likely to have been diagnosed with stage III or IV periodontitis. It has also been suggested that this might be a result of altered neutrophil function and increased production of proinflammatory mediators in older individuals than in younger ones [17]. However, it is not clear how periodontitis is associated with ageing. Tooth extractions are very common in older populations, which may explain the diagnosis of stage IV periodontitis [18]. Other cross-sectional studies have also shown that older adults have a high prevalence and severity of periodontitis, which might be explained by the higher number of teeth retained and the reduction of complete edentulism [19,20].

In addition, in the present study, individuals with generalized, grade C, and stage IV periodontitis demonstrated a significantly higher number of missing teeth (prior to initial periodontal treatment), which would be expected to be severe periodontitis (stage IV) and rapid progression of the disease (grade C) leading to tooth loss. A higher number of teeth lost during supportive periodontal treatment was noted in generalized disease and stage IV periodontitis. This is in agreement with Ravida et al., who demonstrated that higher concomitant staging corresponded to a greater risk for tooth loss due to periodontitis and generalized extent, which was a significant predictor of tooth loss in stage IV or grade C disease [21]. Moreover, the same research group reported that patients with either stage IV or grade C periodontitis exhibited a significantly higher periodontal-related tooth loss [22].

Smoking is a well-established risk factor for periodontal disease onset and progression. It affects the rate of progression and may also increase the conversion from one stage to the next [7]. In the present study, smoking was significantly associated with grade C periodontitis (OR: 2.039, 95% CI: 1.389, 2.995, *p* = 0.01086). Smoking is an environmental factor that has been associated with dysbiotic subgingival microbial communities that increase the risk of periodontal disease development, and a dose-dependent effect has been established in the literature [23,24]. A large NHANES study that included 12,329 adults revealed the significant adverse effect of cigarette smoking on the periodontal tissues as people who reported smoking more than thirty cigarettes per day exhibited an odds ratio of 19.8 to develop periodontitis [24]. Smoking has been associated with a delay in the neutrophils recruitment and mitigation, which compromises the acute immune response [25]. In addition, smoke may shift the balance of neutrophil activities to a more destructive function [26]. Smoking exhibits a two-to-eight-fold increased risk for periodontal attachment loss and/or bone loss, while a significantly inferior treatment outcome is expected in smokers following nonsurgical and surgical periodontal treatment [27,28]. Heavy smokers are associated with a higher risk for attachment and bone loss than light smokers [29].

Multiple sclerosis was found to be associated with the rate of periodontitis progression, and more specifically, with the diagnosis of grade C periodontitis (OR: 60,485.184, 95% CI: 60,485.178, 60,485.190, *p* < 0.001). Multiple sclerosis is one of the most common neurodegenerative disorders, affecting more than 2 million individuals worldwide, and these patients suffer more often by specific oral diseases [30]. Sheu and Lin reported an association between multiple sclerosis and chronic periodontitis, especially in women, and a systematic review of the literature identified evidence supporting the higher risk for periodontal disease development [30,31]. The higher risk for periodontal disease as well as poorer oral hygiene was also reported in another systematic review that included randomized controlled trials, cross-sectional, and cohort studies [32]. Another systematic review that assessed the association between neurodegenerative diseases and periodontitis identified only one eligible case-control study with multiple sclerosis [33]. Patients with multiple sclerosis often encounter changes in bone metabolism that may lead to osteoporosis and osteopenia, which may explain their increased risk of poorer bone quality, bone resorption and bone infections [34,35]. Exacerbation of multiple sclerosis symptoms has been associated with peripheral inflammation due to the presence of periodontal bacteria, demonstrating that patients with periodontitis are also at a higher risk of developing multiple sclerosis [36,37].

To our knowledge, this is the first investigation assessing the association between systemic diseases and smoking with periodontitis diagnosis based on the 2017 World Workshop on the Classification of Periodontal and Peri-Implant Diseases and Conditions. Previous research that has similarly evaluated dental records of patients who attended university dental clinics seeking treatment demonstrated that hypertension, asthma, arthritis, diabetes, and HIV were associated significantly with alveolar bone loss, while in an older population, tobacco use and diabetes were significantly associated with moderate and severe bone loss [38,39]. Moreover, another dental record-based study concluded that the amount of bone loss or the number of missing teeth were significantly associated with vascular disease, heart surgery, vascular surgery, heart attack, thyroid problems, arthritis, and stomach ulcers [40]. In the present investigation, periodontitis diagnoses were utilized rather than alveolar bone loss. Periodontitis diagnosis takes into consideration both clinical and radiographic measures. Another difference between this investigation and previous publications is the inclusion of records of patients from different states in the United States of America, which makes the findings more representative.

A study that utilized data from the 2013–2014 National Healthy and Nutrition Examination Survey (NHANES) in the United States of America examined various chronic diseases, including hypertension, hyperlipidemia, diabetes, arthritis, coronary heart disease, overweight, stroke, asthma, chronic obstructive pulmonary disease, emphysema, chronic bronchitis, cancer, liver condition, thyroid problems, psoriasis, and weak or failing kidneys, and concluded that hypertension and diabetes were only associated with the presence and severity of periodontitis [41]. Although diabetes mellitus is one of the most well-established risk factors for periodontitis onset and progression [42], no association between staging, grading, and extent was identified in the present study. Although individuals who self-reported diabetes were more frequently categorized into grades B (10.9%) and C (10.2%) rather than in grade A (5.9%), as well as in stages III (11.6%) and IV (11.6%) rather than in stages I (7.5%) and II (8.4%), these differences did not reach the level of significance. This might be attributed to the lack of available information regarding diabetes control and glucose levels in the BigMouth Dental Data Repository.

Periodontal disease and inflammatory comorbidities are linked by biologically and clinically consistent mechanisms [9]. Bacteria associated with periodontal disease may disseminate to various tissues, causing inflammatory and functional complications, including endothelial cell dysfunction, bone marrow alterations, gut dysbiosis, and immune suppression, that may initiate or worsen comorbid pathologies [43,44,45,46,47,48,49,50,51,52,53]. In addition, the inflammatory adaptation of hematopoietic progenitors in the bone marrow may explain the connection between multiple chronic inflammatory diseases [54].

It is of paramount importance to consider the inherent limitations of this retrospective cross-sectional study. Although it is impossible to demonstrate causality between risk factors and periodontal disease, it may provide evidence for potential risk indicators that can lead to future studies. Prospective longitudinal studies are required with detailed follow-up to establish cause-and-effect relationships between periodontitis and systemic conditions. All included medical conditions and smoking habits were patient-reported due to the retrospective design of the study. The large number of patient records analyzed in the present investigation minimizes the risk of selection bias. The current findings highlight the association between oral and general health, and can help dental and medical professionals identify patients at risk of disease onset and progression. The cooperation between health care professionals is critical to reduce the occurrence of periodontitis and its comorbidities.

## 5. Conclusions

Within the limitations of this retrospective study that utilized the BigMouth dental data repository, smokers and patients with multiple sclerosis were significantly associated with rapid progression of periodontitis (grade C). None of the examined systemic conditions were associated with the severity and extent of periodontitis. Gender, age, the number of missing teeth, and the number of tooth lost during supportive periodontal treatment were associated with these disease characteristics. Future studies should further investigate the potential causative relationships between these parameters and periodontitis.

## Figures and Tables

**Table 1:** Demographic characteristics of the included population stratified by disease extent.

Characteristics		Overall n = 2069	Generalized n = 1043	Localized n = 1026	*p*-Value *
AGE (mean (SD))		53.81 (14.13)	53.60 (13.76)	54.03 (14.49)	0.494
GENDER (%)	FEMALE	1163 (56.2)	560 (53.7)	603 (58.8)	**0.022**
	MALE	906 (43.8)	483 (46.3)	423 (41.2)	
ETHNICITY (%)	HISPANIC	764 (67.7)	384 (68.3)	380 (67.1)	0.578
	NON-HISPANIC	340 (30.1)	164 (29.2)	176 (31.1)	
	OTHERS	24 (2.1)	14 (2.5)	10 (1.8)	
RACE (%)	WHITE	621 (30.0)	294 (28.2)	327 (31.9)	Reference group
	AMERICAN INDIAN	5 (0.2)	2 (0.2)	3 (0.3)	0.744 ^1^
	ASIAN	196 (9.5)	96 (9.2)	100 (9.7)	0.689 ^1^
	BLACK	391 (18.9)	220 (21.1)	171 (16.7)	**0.006** ^1^
	HISPANIC OR LATINO	719 (34.8)	363 (34.8)	356 (34.7)	0.251 ^1^
	OTHERS	42 (2.0)	21 (2.0)	21 (2.0)	0.739 ^1^
	PACIFIC ISLANDER	2 (0.1)	1 (0.1)	1 (0.1)	0.940 ^1^
	TWO OR MORE	93 (4.5)	46 (4.4)	47 (4.6)	0.703 ^1^
RACE WHITE (%)	NON-WHITE	1392 (67.3)	723 (69.3)	669 (65.2)	**0.051**
	WHITE	677 (32.7)	320 (30.7)	357 (34.8)	
Missing Teeth (median [IQR])		1.00 [0.00, 3.00]	1.00 [0.00, 3.00]	1.00 [0.00, 3.00]	**0.001**
Missing Teeth (mean (SD))		2.12 (2.95)	2.41 (3.26)	1.82 (2.57)	
Tooth Loss during maintenance (median [IQR])		0.00 [0.00, 1.00]	0.00 [0.00, 1.00]	0.00 [0.00, 0.00]	**0.012**
Tooth Loss during maintenance (mean (SD))		0.48 (1.13)	0.56 (1.29)	0.39 (0.93)	

^1^: Based on univariable logistic regression and comparison to White reference group. * The level of significance was set to 0.05 (*p* < 0.05).

**Table 2:** Association between systematic medical conditions and extent.

Patient-Reported Systemic Diseases and Smoking	Generalized n = 1043 (%)	Localized n = 1026 (%)	Odds Ratio (95% CI)	*p* Value *
BLOOD HEMOTOLOGICAL DISORDERS YES	24 (2.3)	25 (2.4)	1.022 (0.576, 1.814)	>0.99
MALIGNANT YES	39 (3.7)	32 (3.1)	0.819 (0.503, 1.333)	>0.99
ANGINA YES	12 (1.2)	10 (1.0)	0.782 (0.334, 1.828)	>0.99
CONGENITAL HEART DISEASE YES	3 (0.3)	5 (0.5)	1.492 (0.352, 6.325)	>0.99
CORONARY HEART DISEASE YES	26 (2.5)	24 (2.3)	1.022 (0.575, 1.817)	>0.99
HISTORY OF ENDOCARDITIS YES	2 (0.2)	1 (0.1)	0.408 (0.037, 4.541)	>0.99
HEART ATTACK YES	13 (1.2)	10 (1.0)	0.856 (0.369, 1.989)	>0.99
HIGH BLOOD PRESSURE YES	423 (40.6)	373 (36.4)	0.832 (0.685, 1.010)	>0.99
IMPLANT DEFIBRILLATOR YES	29 (2.8)	17 (1.7)	0.592 (0.318, 1.101)	>0.99
RHEUMATIC FEVER YES	3 (0.3)	5 (0.5)	1.492 (0.352, 6.325)	>0.99
SMOKING YES	76 (7.3)	41 (4.0)	0.551 (0.372, 0.816)	0.108
ALCOHOL BEVERAGES YES	327 (31.4)	329 (32.1)	1.053 (0.872, 1.271)	>0.99
MARIJUANA USE YES	37 (3.5)	45 (4.4)	1.315 (0.840, 2.059)	>0.99
ANDRENAL GLAND DISORDER YES	1 (0.1)	3 (0.3)	2.573 (0.265,24.933)	>0.99
DIABETES YES	114 (10.9)	92 (9.0)	0.804 (0.598, 1.080)	>0.99
THYROID PROBLEMS YES	75 (7.2)	75 (7.3)	0.926 (0.659, 1.300)	>0.99
GLAUCOMA YES	149 (14.3)	149 (14.5)	0.979 (0.751, 1.276)	>0.99
HIV YES	6 (0.6)	4 (0.4)	0.701 (0.196, 2.505)	>0.99
STD YES	18 (1.7)	11 (1.1)	0.625 (0.293, 1.334)	>0.99
DIALYSIS YES	11 (1.1)	5 (0.5)	0.45 (0.156, 1.305)	>0.99
KIDNEY YES	50 (4.8)	58 (5.7)	1.23 (0.829, 1.825)	>0.99
RENAL FAILURE OR INSUFFICIENCY YES	21 (2.0)	20 (1.9)	0.926 (0.497, 1.724)	>0.99
ARTHRITIS YES	202 (19.4)	198 (19.3)	0.941 (0.745, 1.189)	>0.99
LUPUS YES	4 (0.4)	2 (0.2)	0.502 (0.090, 2.788)	>0.99
OSTEOPOROSIS YES	57 (5.5)	58 (5.7)	0.899 (0.606, 1.334)	>0.99
DEMENTIA YES	6 (0.6)	5 (0.5)	0.778 (0.234, 2.585)	>0.99
ANXIETY YES	77 (7.4)	82 (8.0)	1.078 (0.777, 1.496)	>0.99
DEPRESSION YES	79 (7.6)	92 (9.0)	1.19 (0.866, 1.634)	>0.99
SEIZURE OR EPILEPSY YES	7 (0.7)	12 (1.2)	1.736 (0.679, 4.440)	>0.99
STROKE YES	16 (1.5)	11 (1.1)	0.709 (0.323, 1.558)	>0.99
ORGAN TRANSPLANT YES	6 (0.6)	5 (0.5)	0.782 (0.237, 2.586)	>0.99
ASTHMA YES	4 (0.4)	6 (0.6)	1.39 (0.388, 4.974)	>0.99
CHRONIC BRONCHITIS OR EMPHYSEMA YES	15 (1.4)	5 (0.5)	0.336 (0.121, 0.934)	>0.99
SLEEP APNEA YES	4 (0.4)	2 (0.2)	0.521 (0.095, 2.858)	>0.99
CHRONS DISEASE YES	7 (0.7)	3 (0.3)	0.449 (0.115, 1.760)	>0.99
GASTRO INTESTINAL DISORDERS YES	6 (0.6)	4 (0.4)	0.677 (0.189, 2.422)	>0.99
HEPATITIS YES	45 (4.3)	41 (4.0)	0.914 (0.591, 1.414)	>0.99

* Holm method is used to adjust *p* values for multiple comparison. The level of significance was set to 0.05 (*p* < 0.05).

**Table 3:** Demographic characteristics of the included population stratified by periodontal disease grade.

Characteristics		Overall n = 2069	Grade A n = 341	Grade B n = 1403	Grade C n = 325	*p*-Value *
AGE (mean (SD))		53.81 (14.13)	53.36 (14.25)	54.32 (14.21)	52.11 (13.49)	**0.032**
GENDER (%)	Female	1163 (56.2)	198 (58.1)	779 (55.5)	186 (57.2)	0.643
	Male	906 (43.8)	143 (41.9)	624 (44.5)	139 (42.8)	
ETHNICITY (%)	HISPANIC	764 (67.7)	127 (66.1)	527 (68.0)	110 (68.3)	0.168
	NON HISPANIC	340 (30.1)	56 (29.2)	236 (30.5)	48 (29.8)	
	OTHERS	24 (2.1)	9 (4.7)	12 (1.5)	3 (1.9)	
RACE (%)	WHITE	621 (30.0)	115 (33.7)	400 (28.5)	106 (32.6)	Reference group
	AMERICAN INDIAN	5 (0.2)	0 (0.0)	2 (0.1)	3 (0.9)	**0.017** ^1^
	ASIAN	196 (9.5)	26 (7.6)	133 (9.5)	37 (11.4)	0.127 ^1^
	BLACK	391 (18.9)	58 (17.0)	279 (19.9)	54 (16.6)	0.896 ^1^
	HISPANIC OR LATINO	719 (34.8)	112 (32.8)	499 (35.6)	108 (33.2)	0.765 ^1^
	OTHERS	42 (2.0)	10 (2.9)	28 (2.0)	4 (1.2)	0.154 ^1^
	PACIFIC ISLANDER	2 (0.1)	1 (0.3)	1 (0.1)	0 (0.0)	0.225 ^1^
	TWO OR MORE	93 (4.5)	19 (5.6)	61 (4.3)	13 (4.0)	0.425 ^1^
RACE (%)	NON-WHITE	1392 (67.3)	210 (61.6)	968 (69.0)	214 (65.8)	**0.027**
	WHITE	677 (32.7)	131 (38.4)	435 (31.0)	111 (34.2)	
Missing Teeth (median [IQR])		1.00 [0.00, 3.00]	1.00 [0.00, 3.00]	1.00 [0.00, 3.00]	2.00 [0.00, 4.00]	**0.019**
Missing Teeth (mean (SD))		2.12 (2.95)	1.92 (2.64)	2.02 (2.80)	2.74 (3.73)	
Tooth Loss during maintenance (median [IQR])		0.00 [0.00, 1.00]	0.00 [0.00, 1.00]	0.00 [0.00, 1.00]	0.00 [0.00, 1.00]	0.422
Tooth Loss during maintenance (mean (SD))		0.48 (1.13)	0.44 (1.02)	0.45 (1.09)	0.61 (1.36)	

^1^: Based on univariable proportional odds regression and comparison to Whites reference group. * The level of significance was set to 0.05 (*p* < 0.05).

**Table 4:** Association between systematic medical conditions and periodontal disease rate of progression (grade).

Patient-Reported Systemic Diseases and Smoking	Grade A n = 341 (%)	Grade B n = 1403 (%)	Grade C n = 325 (%)	Odds Ratio (95% CI)	*p* Value *
BLOOD HEMOTOLOGICAL DISORDERS YES	8 (2.3)	32 (2.3)	9 (2.8)	1.116 (0.609, 2.044)	>0.99
MALIGNANT YES	13 (3.8)	47 (3.3)	11 (3.4)	0.97 (0.584, 1.610)	>0.99
ANGINA YES	6 (1.8)	15 (1.1)	1 (0.3)	0.489 (0.211, 1.132)	>0.99
CONGENITAL HEART DISEASE YES	0 (0.0)	8 (0.6)	0 (0.0)	1.138 (0.297, 4.362)	>0.99
CORONARY HEART DISEASE YES	9 (2.6)	33 (2.4)	8 (2.5)	0.94 (0.515, 1.716)	>0.99
HISTORY OF ENDOCARDITIS YES	0 (0.0)	3 (0.2)	0 (0.0)	1.234 (0.138, 11.041)	>0.99
HEART ATTACK YES	2 (0.6)	17 (1.2)	4 (1.2)	1.372 (0.592, 3.178)	>0.99
HIGH BLOOD PRESSURE YES	131 (38.4)	560 (39.9)	105 (32.3)	0.847 (0.692, 1.038)	>0.99
IMPLANT DEFIBRILLATOR YES	5 (1.5)	33 (2.4)	8 (2.5)	1.357 (0.733, 2.515)	>0.99
RHEUMATIC FEVER YES	0 (0.0)	8 (0.6)	0 (0.0)	1.138 (0.297, 4.362)	>0.99
SMOKING YES	9 (2.6)	78 (5.6)	30 (9.2)	2.014 (1.370, 2.960)	**0.014**
COCAINE USE YES	0 (0.0)	1 (0.1)	0 (0.0)	1.099 (0.025, 48.587)	>0.99
ALCOHOL BEVERAGES YES	110 (32.3)	456 (32.5)	90 (27.7)	0.891 (0.732, 1.085)	>0.99
MARIJUANA USE YES	13 (3.8)	50 (3.6)	19 (5.8)	1.36 (0.847, 2.186)	>0.99
ANDRENAL GLAND DISORDER YES	1 (0.3)	3 (0.2)	0 (0.0)	0.487 (0.071, 3.341)	>0.99
DIABETES YES	20 (5.9)	153 (10.9)	33 (10.2)	1.35 (0.999, 1.825)	>0.99
THYROID PROBLEMS YES	36 (10.6)	97 (6.9)	17 (5.2)	0.643 (0.452, 0.916)	0.533
GLAUCOMA YES	55 (16.1)	208 (14.8)	35 (10.8)	0.791 (0.601, 1.041)	>0.99
HIV YES	3 (0.9)	5 (0.4)	2 (0.6)	0.706 (0.181, 2.757)	>0.99
STD YES	3 (0.9)	21 (1.5)	5 (1.5)	1.318 (0.617, 2.816)	>0.99
DIALYSIS YES	1 (0.3)	12 (0.9)	3 (0.9)	1.666 (0.614, 4.519)	>0.99
KIDNEY YES	17 (5.0)	72 (5.1)	19 (5.8)	1.138 (0.753, 1.721)	>0.99
RENAL FAILURE OR INSUFFICIENCY YES	4 (1.2)	27 (1.9)	10 (3.1)	1.925 (1.016, 3.649)	>0.99
ARTHRITIS YES	78 (22.9)	277 (19.7)	45 (13.8)	0.708 (0.555, 0.903)	0.204
LUPUS YES	1 (0.3)	3 (0.2)	2 (0.6)	1.891 (0.346, 10.347)	>0.99
OSTEOPOROSIS YES	26 (7.6)	77 (5.5)	12 (3.7)	0.666 (0.443, 1.000)	>0.99
DEMENTIA YES	1 (0.3)	9 (0.6)	1 (0.3)	1.084 (0.326, 3.608)	>0.99
ANXIETY YES	28 (8.2)	106 (7.6)	25 (7.7)	0.94 (0.669, 1.322)	>0.99
DEPRESSION YES	30 (8.8)	120 (8.6)	21 (6.5)	0.822 (0.593, 1.139)	>0.99
PARKINSON DISEASE YES	0 (0.0)	1 (0.1)	3 (0.9)	14.881 (1.539, 143.913)	0.709
MULTIPLE SCLEROSIS YES	0 (0.0)	0 (0.0)	1 (0.3)	60,021.028 (60,021.023,60,021.033)	**<0.001**
SEIZURE OR EPILEPSY YES	5 (1.5)	11 (0.8)	3 (0.9)	0.682 (0.259, 1.791)	>0.99
STROKE YES	3 (0.9)	19 (1.4)	5 (1.5)	1.38 (0.627, 3.040)	>0.99
ORGAN TRANSPLANT YES	2 (0.6)	6 (0.4)	3 (0.9)	1.586 (0.443, 5.685)	>0.99
ASTHMA YES	5 (1.5)	4 (0.3)	1 (0.3)	0.239 (0.067, 0.848)	0.935
CHRONIC BRONCHITIS OR EMPHYSEMA YES	3 (0.9)	13 (0.9)	4 (1.2)	1.242 (0.492, 3.135)	>0.99
SLEEP APNEA YES	0 (0.0)	5 (0.4)	1 (0.3)	1.868 (0.388, 8.982)	>0.99
CHRONS DISEASE YES	1 (0.3)	7 (0.5)	2 (0.6)	1.37 (0.373, 5.034)	>0.99
GASTRO INTESTINAL DISORDERS YES	2 (0.6)	7 (0.5)	1 (0.3)	0.677 (0.186, 2.464)	>0.99
HEPATITIS YES	14 (4.1)	63 (4.5)	9 (2.8)	0.847 (0.541, 1.325)	>0.99

* Holm method is used to adjust *p* values for multiple comparison. Bold denotes significance. The level of significance was set to 0.05 (*p* < 0.05).

**Table 5:** Demographic characteristics of the included population stratified by periodontal disease severity (stage).

Characteristics		Overall n = 2069	Stage I n = 294	Stage II n = 677	Stage III n = 960	Stage IV n = 138	*p*-Value *
AGE (mean (SD))		53.81 (14.13)	51.21 (15.72)	52.17 (14.28)	55.40 (13.56)	56.43 (11.73)	**<0.001**
GENDER (%)	Female	1163 (56.2)	183 (62.2)	403 (59.5)	503 (52.4)	74 (53.6)	**0.004**
	Male	906 (43.8)	111 (37.8)	274 (40.5)	457 (47.6)	64 (46.4)	
ETHNICITY (%)	HISPANIC	764 (67.7)	118 (65.2)	251 (65.9)	346 (69.6)	49 (71.0)	0.732
	NON-HISPANIC	340 (30.1)	59 (32.6)	122 (32.0)	139 (28.0)	20 (29.0)	
	OTHERS	24 (2.1)	4 (2.2)	8 (2.1)	12 (2.4)	0 (0.0)	
RACE (%)	WHITE	621 (30.0)	95 (32.3)	203 (30.0)	291 (30.3)	32 (23.2)	Reference group
	AMERICAN INDIAN	5 (0.2)	1 (0.3)	1 (0.1)	3 (0.3)	0 (0.0)	0.955 ^1^
	ASIAN	196 (9.5)	22 (7.5)	60 (8.9)	101 (10.5)	13 (9.4)	0.087 ^1^
	BLACK	391 (18.9)	40 (13.6)	135 (19.9)	179 (18.6)	37 (26.8)	**0.037** ^1^
	HISPANIC OR LATINO	719 (34.8)	112 (38.1)	233 (34.4)	325 (33.9)	49 (35.5)	0.805 ^1^
	OTHERS	42 (2.0)	7 (2.4)	11 (1.6)	22 (2.3)	2 (1.4)	0.709 ^1^
	PACIFIC ISLANDER	2 (0.1)	1 (0.3)	1 (0.1)	0 (0.0)	0 (0.0)	0.125 ^1^
	TWO OR MORE	93 (4.5)	16 (5.4)	33 (4.9)	39 (4.1)	5 (3.6)	0.450 ^1^
RACE WHITE (%)	NON-WHITE	1392 (67.3)	189 (64.3)	451 (66.6)	649 (67.6)	103 (74.6)	0.189
	WHITE	677 (32.7)	105 (35.7)	226 (33.4)	311 (32.4)	35 (25.4)	
Missing Teeth (median [IQR])		1.00 [0.00, 3.00]	1.00 [0.00, 3.00]	1.00 [0.00, 3.00]	1.00 [0.00, 3.00]	3.00 [0.00, 6.00]	**<0.001**
Missing Teeth (mean (SD))		2.12 (2.95)	1.81 (2.55)	1.78 (2.55)	2.14 (2.89)	4.29 (4.65)	
Tooth Loss during maintenance (median [IQR])		0.00 [0.00, 1.00]	0.00 [0.00, 0.00]	0.00 [0.00, 0.00]	0.00 [0.00, 1.00]	0.00 [0.00, 2.00]	**<0.001**
Tooth Loss during maintenance (mean (SD))		0.48 (1.13)	0.37 (0.90)	0.34 (0.87)	0.50 (1.17)	1.16 (1.91)	

^1^: Based on univariable proportional odds regression and comparison to White s reference group. * The level of significance was set to 0.05 (*p* < 0.05).

**Table 6:** Association between systematic medical conditions and stage.

Patient-Reported Systemic Diseases and Smoking	Stage I n = 294	Stage II n = 677	Stage III n = 960	Stage IV n = 138	Odds Ratio (95% CI)	*p* Value *
BLOOD HEMOTOLOGICAL DISORDERS YES	2 (0.7)	20 (3.0)	19 (2.0)	8 (5.8)	1.469 (0.850, 2.539)	>0.99
MALIGNANT YES	10 (3.4)	23 (3.4)	36 (3.8)	2 (1.4)	0.707 (0.454, 1.103)	>0.99
ANGINA YES	8 (2.7)	6 (0.9)	6 (0.6)	2 (1.4)	0.391 (0.169, 0.906)	>0.99
CONGENITAL HEART DISEASE YES	0 (0.0)	0 (0.0)	7 (0.7)	1 (0.7)	4.587 (1.284, 16.388)	0.723
CORONARY HEART DISEASE YES	8 (2.7)	17 (2.5)	22 (2.3)	3 (2.2)	0.568 (0.332, 0.973)	>0.99
HISTORY OF ENDOCARDITIS YES	0 (0.0)	3 (0.4)	0 (0.0)	0 (0.0)	0.335 (0.058, 1.952)	>0.99
HEART ATTACK YES	5 (1.7)	6 (0.9)	11 (1.1)	1 (0.7)	0.54 (0.247, 1.182)	>0.99
HIGH BLOOD PRESSURE YES	97 (33.0)	252 (37.2)	381 (39.7)	66 (47.8)	0.995 (0.830, 1.192)	>0.99
IMPLANT DEFIBRILLATOR YES	4 (1.4)	16 (2.4)	22 (2.3)	4 (2.9)	0.889 (0.510, 1.549)	>0.99
RHEUMATIC FEVER YES	0 (0.0)	0 (0.0)	7 (0.7)	1 (0.7)	4.587 (1.284, 16.388)	0.723
SMOKING YES	13 (4.4)	35 (5.2)	54 (5.6)	15 (10.9)	1.456 (1.013, 2.094)	>0.99
COCAINE USE YES	0 (0.0)	1 (0.1)	0 (0.0)	0 (0.0)	0.475 (0.023, 9.885)	>0.99
ALCOHOL BEVERAGES YES	93 (31.6)	217 (32.1)	300 (31.2)	46 (33.3)	0.936 (0.785, 1.116)	>0.99
MARIJUANA USE YES	12 (4.1)	25 (3.7)	39 (4.1)	6 (4.3)	1.101 (0.725, 1.672)	>0.99
ANDRENAL GLAND DISORDER YES	0 (0.0)	2 (0.3)	2 (0.2)	0 (0.0)	1.168 (0.217, 6.298)	>0.99
DIABETES YES	22 (7.5)	57 (8.4)	111 (11.6)	16 (11.6)	1.211 (0.918, 1.598)	>0.99
THYROID PROBLEMS YES	27 (9.2)	52 (7.7)	63 (6.6)	8 (5.8)	0.733 (0.536, 1.004)	>0.99
GLAUCOMA YES	41 (13.9)	90 (13.3)	146 (15.2)	21 (15.2)	0.857 (0.668, 1.100)	>0.99
HIV YES	4 (1.4)	2 (0.3)	2 (0.2)	2 (1.4)	0.386 (0.098, 1.516)	>0.99
STD YES	6 (2.0)	9 (1.3)	11 (1.1)	3 (2.2)	0.867 (0.430, 1.748)	>0.99
DIALYSIS YES	1 (0.3)	10 (1.5)	4 (0.4)	1 (0.7)	0.77 (0.333, 1.781)	>0.99
KIDNEY YES	14 (4.8)	39 (5.8)	51 (5.3)	4 (2.9)	0.772 (0.539, 1.104)	>0.99
RENAL FAILURE OR INSUFFICIENCY YES	4 (1.4)	18 (2.7)	17 (1.8)	2 (1.4)	0.841 (0.484, 1.463)	>0.99
ARTHRITIS YES	58 (19.7)	139 (20.5)	176 (18.3)	27 (19.6)	0.75 (0.603, 0.934)	0.391
LUPUS YES	2 (0.7)	2 (0.3)	2 (0.2)	0 (0.0)	0.326 (0.072, 1.473)	>0.99
OSTEOPOROSIS YES	16 (5.4)	32 (4.7)	57 (5.9)	10 (7.2)	1.103 (0.757, 1.607)	>0.99
DEMENTIA YES	1 (0.3)	6 (0.9)	3 (0.3)	1 (0.7)	0.566 (0.193, 1.660)	>0.99
ANXIETY YES	31 (10.5)	56 (8.3)	61 (6.4)	11 (8.0)	0.759 (0.559, 1.029)	>0.99
DEPRESSION YES	32 (10.9)	59 (8.7)	69 (7.2)	11 (8.0)	0.758 (0.564, 1.018)	>0.99
PARKINSON DISEASE YES	0 (0.0)	0 (0.0)	1 (0.1)	3 (2.2)	36.366 (3.641, 363.203)	0.088
MULTIPLE SCLEROSIS YES	0 (0.0)	0 (0.0)	1 (0.1)	0 (0.0)	4.289 (0.129, 142.762)	>0.99
SEIZURE OR EPILEPSY YES	1 (0.3)	7 (1.0)	9 (0.9)	2 (1.4)	1.449 (0.626, 3.354)	>0.99
STROKE YES	5 (1.7)	9 (1.3)	11 (1.1)	2 (1.4)	0.647 (0.312, 1.341)	>0.99
ORGAN TRANSPLANT YES	1 (0.3)	4 (0.6)	4 (0.4)	2 (1.4)	1.392 (0.435, 4.450)	>0.99
ASTHMA YES	2 (0.7)	5 (0.7)	3 (0.3)	0 (0.0)	0.403 (0.135, 1.202)	>0.99
CHRONIC BRONCHITIS OR EMPHYSEMA YES	5 (1.7)	6 (0.9)	6 (0.6)	3 (2.2)	0.664 (0.274, 1.609)	>0.99
SLEEP APNEA YES	0 (0.0)	3 (0.4)	3 (0.3)	0 (0.0)	1.04 (0.268, 4.038)	>0.99
CHRONS DISEASE YES	2 (0.7)	1 (0.1)	5 (0.5)	2 (1.4)	2.165 (0.556, 8.435)	>0.99
GASTRO INTESTINAL DISORDERS YES	3 (1.0)	2 (0.3)	4 (0.4)	1 (0.7)	0.726 (0.206, 2.562)	>0.99
HEPATITIS YES	12 (4.1)	19 (2.8)	50 (5.2)	5 (3.6)	1.233 (0.813, 1.869)	>0.99

* Holm method is used to adjust *p* values for multiple comparison. The level of significance was set to 0.05 (*p* < 0.05).

## Data Availability

The datasets used and/or analysed during the current study are available from the corresponding author on reasonable request.
